# *Mycobacterium tuberculosis* infection of host cells in space and time

**DOI:** 10.1093/femsre/fuz006

**Published:** 2019-03-27

**Authors:** Claudio Bussi, Maximiliano G Gutierrez

**Affiliations:** Host-pathogen interactions in tuberculosis laboratory, The Francis Crick Institute, 1 Midland Road, London, NW1 1AT, United Kingdom; Host-pathogen interactions in tuberculosis laboratory, The Francis Crick Institute, 1 Midland Road, London, NW1 1AT, United Kingdom

**Keywords:** *Mycobacterium tuberculosis*, macrophage, phagosome, autophagy, Tuberculosis

## Abstract

Tuberculosis (TB) caused by the bacterial pathogen *Mycobacterium tuberculosis* (Mtb) remains one of the deadliest infectious diseases with over a billion deaths in the past 200 years (Paulson 2013). TB causes more deaths worldwide than any other single infectious agent, with 10.4 million new cases and close to 1.7 million deaths in 2017. The obstacles that make TB hard to treat and eradicate are intrinsically linked to the intracellular lifestyle of Mtb. Mtb needs to replicate within human cells to disseminate to other individuals and cause disease. However, we still do not completely understand how Mtb manages to survive within eukaryotic cells and why some cells are able to eradicate this lethal pathogen. Here, we summarise the current knowledge of the complex host cell-pathogen interactions in TB and review the cellular mechanisms operating at the interface between Mtb and the human host cell, highlighting the technical and methodological challenges to investigating the cell biology of human host cell-Mtb interactions.

## Teaser

The review describes the complex dynamics underlying the interactions between *Mycobacterium tuberculosis* and the human host cell with an emphasis on the cell biology approaches and potential challenges to study these processes.

## FROM TISSUES TO PHAGOSOMES: THE ENVIRONMENTS MTB FACES IN THE HOST

The tubercle bacillus spreads from person-to-person almost exclusively by aerosolized particles. The size of infectious droplets from Mtb infected patients ranges from 0.65 (small) to > 7.0 μm (medium–large) (Fennelly and Jones-Lopez 2015). While small Mtb aerosol particles are expected to transit the nasopharyngeal or tracheobronchial region to be deposited in the distal airways, larger particles can be trapped in the upper airway or oropharynx where they can potentially lead to Tuberculosis (TB) of the oropharynx or cervical lymph nodes (Laal 2012; Fennelly and Jones-Lopez 2015).

**Figure 1.**
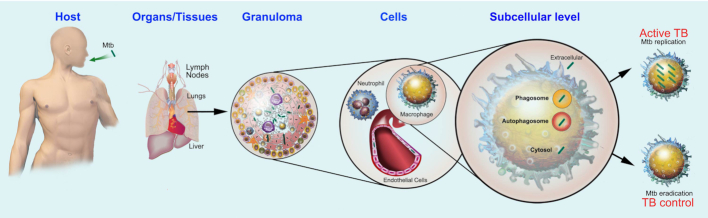
Host cells and environments for Mtb. TB is transmitted from an infected to a susceptible person in airborne particles, called droplet nuclei. Transmission occurs when a person inhales droplet nuclei containing Mtb, and the droplet nuclei traverse the mouth or nasal passages, upper respiratory tract and bronchi to reach the alveoli of the lungs. Although TB is primarily a lung infection, it can also disseminate to other organs and tissues. Once Mtb colonises the host, an inflammatory cellular infiltrate to these sites could trigger, most prominently in the lungs, the formation of granulomas, whose are cellular aggregates constituted by macrophages, multinucleated giant cells, epithelioid and foamy cells, granulocytes and lymphocytes. Mtb can infect several cell types, including neutrophils, macrophages and endothelial cells. Once internalized by the cell, Mtb can reside in different cellular compartments such as, phagosomes and autophagosomes and, if disrupting these organelles, Mtb can also gain access to the cytosol. The host will utilise several cellular and immunological mechanisms to control the infection that will compete with a broad range of Mtb evasion and virulence strategies, that if successful, will allow bacterial survival and replication.

Once in the lower respiratory tract, Mtb is primarily phagocytosed by macrophages and dendritic cells. In addition to macrophages and dendritic cells, studies analysing the sputum of TB patients identified neutrophils as the predominant phagocytic cell infected with Mtb (Eum *et al*. 2010). There is also evidence that Mtb is also found in non-myelocytic cells of TB patients (Lerner, Borel and Gutierrez 2015; Randall *et al*. 2015; Ganbat *et al*. 2016) (Fig. 1). The bacilli that manage to pass through the upper airways will be delivered to the alveoli. The alveoli consist of type I and II epithelial cells with a number of other immune cells such as alveolar macrophages, dendritic cells and neutrophils (Lerner, Borel and Gutierrez 2015). Infection of type II alveolar epithelial cells by Mtb has been extensively studied *in vitro* and DNA from Mtb has been detected within these cells in post-mortem studies (Hernández-Pando *et al*. 2000). Among the specialised epithelial cells of the upper airway, Mtb invades the cells known as a microfold cell (M cell) in the lungs of mice to initiate infection (Nair *et al*. 2016). Infected cells will trigger a local inflammatory response that will attract immune cells into the site of infection. These cellular aggregates, that contain many cell types, form the granulomas, the pathological hallmarks of TB. These granulomatous structures represent a complex environment for Mtb, very different from the one associated with healthy tissue. Although the mechanism is not entirely clear, these single lesion environments are likely very permissive for bacterial growth as well as impairing dissemination (Rubin 2009).

Outside the lungs, Mtb can virtually disseminate to any organ, with the lymphatics and lymph nodes being the main sites of extrapulmonary TB (Behr and Waters 2014). In this context, aerosol infection of mice lacking M cells have significantly delayed dissemination to lymph nodes and reduced mortality, suggesting that M cells contribute to dissemination and disease progression (Nair *et al*. 2016). In the lymph nodes of patients with extrapulmonary TB, Mtb was found to infect lymphatic endothelial cells (LEC) (Lerner *et al*. 2016; Lerner *et al*. 2017). LEC are cells that line the lymphatic vessels and therefore the first ones to encounter bacteria that disseminates through lymphatics. Because these cells are non-motile, they could provide a niche for Mtb that facilitates persistent infection in lymph nodes (Lerner *et al*. 2016). Moreover, the adipose tissue (Beigier-Bompadre *et al*. 2017) and bone marrow (Mayito *et al*. 2019) have been proposed as extrapulmonary niches where the tubercle bacillus could persist for long periods of time modulating the local tissue environment.

Collectively, *in vitro* and *in vivo* evidence show that Mtb infects different cell types during infection, encountering distinct environments within these cells (Fig. 1). The impact of these environments for Mtb on TB dissemination or disease progression remain unclear. One of the main arguments against a role for non-myelocytic cells during TB is based on the idea that only a minor proportion of these cells are potentially infected. However, it is still unclear whether a minor proportion of infected non-myelocytic cells could affect the outcome of active TB. The evidence arguing for several cell types playing a role during TB needs to be carefully considered and not necessarily seen as a potential bystander effect. How different specific cell-type environments affect Mtb localisation and survival (Fig. 1) is poorly characterised and these differences could have a profound impact during disease dissemination, progression and resolution. For example, Mtb must face very different intracellular niches in macrophages when compared to neutrophils or endothelial cells (Fig. 1).

## THE SPACE

### In a membrane-bound compartment: tight and spacious phagosomes

**Figure 2.**
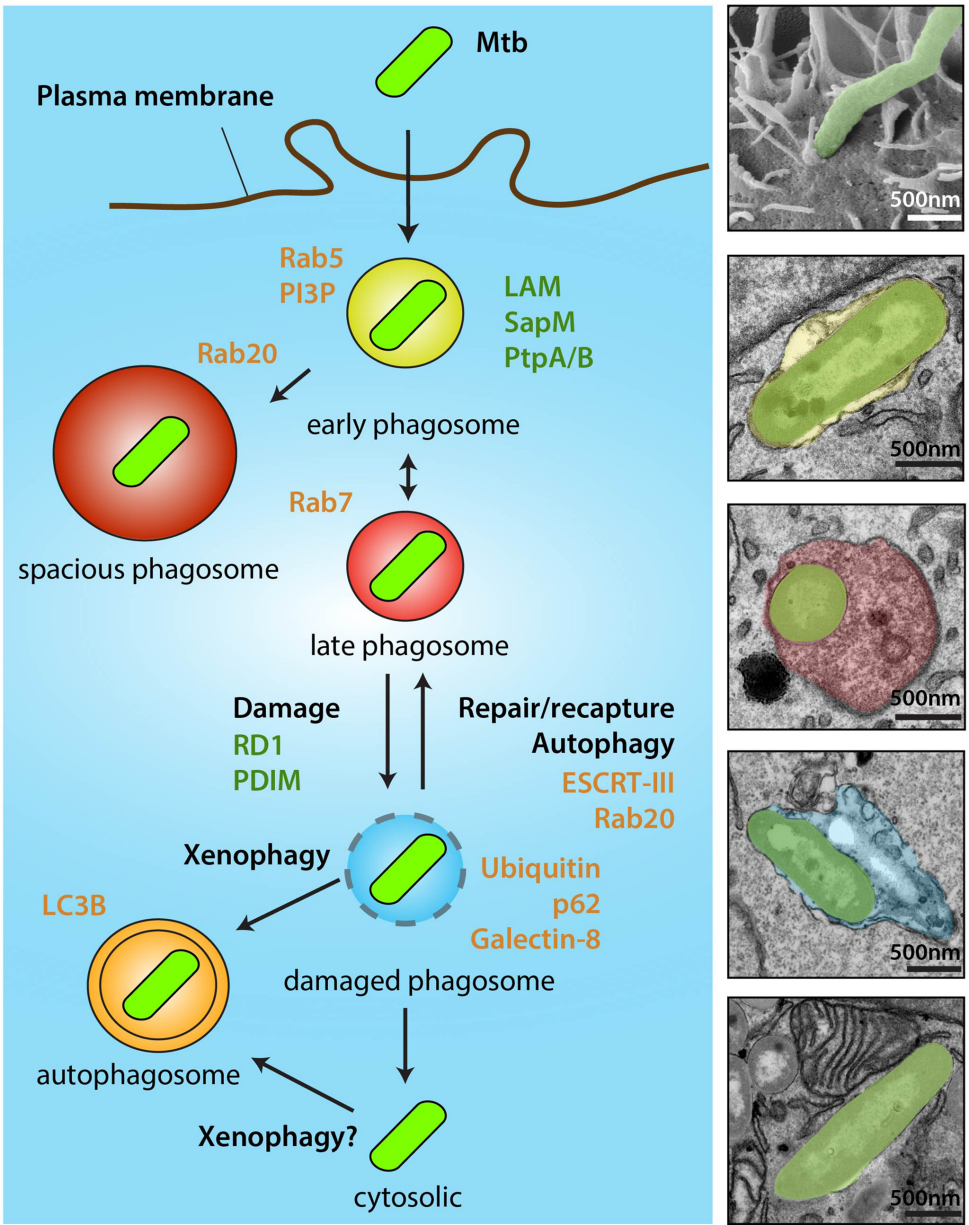
The space: localisation of Mtb within human cells. Once Mtb is phagocytosed by macrophages, Mtb will reside in early phagosomes and inhibition of phagosome-lysosome fusion is central to the survival of Mtb. Mtb uses different strategies to interfere with phagosome maturation. Some Mtb-containing phagosomes may fuse with late endosomes resulting in some mycobacteria localised in late endosomes. On the other hand, Mtb may disrupt the phagosomal membrane and escape to the cytosol. In the example shown, membrane damage is partially observed although artefacts from chemical fixation cannot be excluded. These different stages are not stable but highly dynamic. The panel on the right shows representative EM images of Mtb-infected human macrophages illustrating the different cellular compartments where Mtb can localise/reside within host cells. Host molecules associated with the different localisation stages are shown in orange and Mtb factors implicated in the process shown in green.

Mtb is internalised by phagocytosis in phagocytic cells such as macrophages (Fig. 2), dendritic cells and neutrophils. This actin-dependent process is regulated by many receptors and depends on the cell type. Phagocytosis of Mtb by macrophages is mediated via an array of different receptor molecules, including dectin-1, the complement receptor 3, Toll-like receptors, mannose receptor, the dendritic cell-specific intercellular adhesion molecule (ICAM)-3-grabbing nonintegrin (DC-SIGN), Fc receptors, scavenger receptors and CD14 (Pieters 2008; Schafer *et al*. 2009).

Although the relevance during human TB is unclear, numerous studies performed mostly *in vitro*, showed the internalization of Mtb by non-phagocytic cells such as type II alveolar epithelial cells, fibroblasts and stem cells. This process involves heparin-binding hemagglutinin, TLRs, surfactant proteins and complement and scavenger receptors (Lerner, Borel and Gutierrez 2015). In human tissue from TB patients, mycobacteria were also found in adipocytes, LEC and stem cells, suggesting these cellular environments are permissive for Mtb survival and subsequent reactivation (Neyrolles *et al*. 2006; Das *et al*. 2013; Lerner *et al*. 2016; Tornack *et al*. 2017).

While *in vitro* studies have demonstrated clear roles for particular receptor(s), it is more than likely that *in vivo*, Mtb is not internalized by macrophages using a single receptor-mediated pathway. Mycobacteria display numerous and diverse ligands on their surface which might either engage multiple receptors of multiple types simultaneously, or might bind to those receptors that are available on a particular cell. Moreover, mycobacterial surface molecules might also function to impair the formation of adequate receptor clustering (Schafer *et al*. 2009; Liu, Liu and Ge 2017). Importantly, the type of receptor engaged in phagocytosis can influence downstream signalling and influence phagosomal fate at the single phagosome level, independently of other phagosomes within the same cell (Hoffmann *et al*. 2012).

After Mtb is internalised in a phagosome, these phagosomes will subsequently interact with early and late endocytic organelles as well as other intracellular organelles (see below) in a process referred to ‘phagosome maturation’ (Fig. 2). These interactions are very dynamic and rapidly change the membrane and luminal composition of the phagosome, with the primary aim of restricting the growth of the internalised pathogen.

Mtb has been considered to be a classical bacterial pathogen that primarily resides within phagosomes of macrophages (reviewed in (Russell 2001; Vergne *et al*. 2004a; Pieters 2008)). Pioneering studies using electron microscopy (EM) from D'Arcy Hart and collaborators localised Mtb in phagosomes (Armstrong and Hart 1971). Other EM studies performed in the 80’s led to conclusions that differed from D'Arcy Hart's original observations and localised Mtb outside the phagosome in the cytosol of macrophages (see ‘*In the cytosol*’ section below). Notably, in the studies from D'Arcy Hart the possibility that Mtb phagosomes were fusing with late endocytic organelles (herein referred as lysosomes for simplicity) was observed (Armstrong and Hart 1975). Phagosomes containing Mtb or a related pathogen, *M. avium* in human macrophages were non-acidic suggesting a defect in phagosomal acidification (Crowle *et al*. 1991). In human monocytes, Mtb was mostly localised in phagosomes that do not fuse with late endosomes/lysosomes (Clemens and Horwitz 1995) but display some interactions with early endosomes and remained accessible to transferrin (Clemens and Horwitz 1996). More recent EM studies confirmed that a significant proportion of mycobacteria localise in membrane bound compartments (Jordao *et al*. 2008). Strikingly, EM localisation in alveolar macrophages isolated from a patient with active TB in Malawi showed membrane-bound bacteria which localised sometimes in large vacuoles containing multiple bacilli (Russell, Mwandumba and Rhoades 2002).

Because of the observed deficient luminal acidification, efforts focused mostly on finding the mechanisms by which Mtb remained in a non-acidic compartment and ‘arrested’ phagosome maturation. Research in this area contributed to the idea that Mtb interferes with the maturation of phagosomes to survive within macrophages and a striking feature of the Mtb phagosome were its defective recruitment of the vacuolar ATPase (vATPase) and reduced levels of mature lysosomal hydrolases (Sturgill-Koszycki *et al*. 1994). The mechanism of the vATPase exclusion by Mtb phagosomes is associated to the STAT-5-mediated expression of cytokine-inducible SH2-containing protein, which selectively targets the vATPase for ubiquitination and degradation (Queval *et al*. 2017).

As our understanding of the molecular machinery controlling phagosome and endosome maturation gradually emerged, this knowledge was applied to the study of the Mtb phagosome. Mechanistic reports of the process leading to the mycobacterial phagosome maturation arrest began with the study of endocytic Rab GTPases, which regulate intracellular trafficking and maintain the identity of cellular organelles (Vergne *et al*. 2004a). Early studies indicated that a functional block occurred between the maturation stages controlled by the small GTPase Rab5 (early endosomal) and Rab7 (late endosomal/lysosomal) (Via *et al*. 1997). Rab5 accumulated on mycobacterial phagosomes, whereas Rab7 was not recruited to the phagosomes (Via *et al*. 1997). A complementary study found that while Rab5 effectors are recruited to the mycobacterial phagosome, the Rab5 effector early endosomal autoantigen 1 (EEA1) associated with the process of membrane fusion was excluded (Fratti *et al*. 2001). EEA1 directly interacts with Rab5 and its recruitment and association with endosomal membranes are strengthened by the binding of its FYVE domain to phosphatidylinositol 3‐phosphate (PI3P) (Simonsen *et al*. 1998). PI3P is generated on endosomal membranes by the action of the class III phosphatidylinositol 3‐kinase (PI3K) and Rab5 effector, hVPS34 (Christoforidis *et al*. 1999). Thus, the impaired recruitment of EEA1 observed in mycobacterial phagosomes provided a mechanistic link between PI3K/PI3P and the mycobacterial phagosome maturation arrest (Fratti *et al*. 2001).

Experiments showing that mycobacteria-containing compartments access transferrin-bound iron (Sturgill-Koszycki, Schaible and Russell 1996), accumulation of transferrin receptor on mycobacterial phagosomes (Clemens and Horwitz 1996) and accessibility to glycosphingolipids (Russell, Dant and Sturgill-Koszycki 1996) supported the idea that the mycobacterial phagosome is not a static organelle. For instance, the survival of Mtb is associated with Coronin 1 (also known as TACO), which transiently accumulates on phagosomal membranes (Ferrari *et al*. 1999; Mori, Mode and Pieters 2018) and is actively retained by living mycobacteria residing inside phagosomes. Coronin 1 is responsible for the activation of the Ca^2+^-dependent phosphatase calcineurin, which in turn impairs the delivery of mycobacteria to phagolysosomes (Jayachandran *et al*. 2007). On the other hand, a number of reports suggested that mycobacteria inhibit actin polymerization (Guerin and de Chastellier 2000; Anes *et al*. 2003).

In addition, the composition, the movement and the role of metals and trace elements have an impact on the biology of the microbial phagosome (Soldati and Neyrolles 2012). For instance, the mycobacterial phagosome is readily emptied of phosphorus, sulphur and chlorine upon inflammatory stimulation (Wagner *et al*. 2005). However, if phosphate depletion from the vacuole is associated to the induction of a dormancy program previously reported in mycobacteria cultured in phosphate‐limiting conditions (Rifat, Bishai and Karakousis 2009), remains to be determined.

Mtb also directly alters host signalling through the secretion of phosphatases, thereby shutting down critical cellular processes and promoting its survival within macrophages (Saleh and Belisle 2000; Bach *et al*. 2008; Wong *et al*. 2011; Wong *et al*. 2013). Mtb-secreted protein- and lipid-phosphatases protein-tyrosine phosphatase A and B (PtpA and PtpB), and secreted acid phosphatase M have been shown to contribute to Mtb pathogenicity by disrupting the endocytic pathway and promoting phagosome maturation arrest (reviewed in (Wong, Chao and Av-Gay 2013)). These secreted enzymes represent a good strategy to target Mtb and avoiding the development of drug resistance. For example, selective inhibitors of MptpB alter Mtb phagosomal PI3P dynamics that correlates with increased bacterial control in macrophages and guinea pigs (Vickers *et al*. 2018).

The above mentioned mechanistic studies of phagosome maturation ‘arrest’ are important for our understanding of the phagosomal stage of mycobacteria. Conclusions from these studies were generally based on limited quantitative imaging data and performed in host cells from different sources and species, making comparisons difficult. The postulated mechanisms were mostly based on data generated using avirulent mycobacteria such as the vaccine strain Bacillus Calmette-Guerin (BCG) and in many cases using Western blot analysis of mycobacteria-containing phagosomes, a method that is prone to significant contamination with other organelles. In consequence, and mostly due to the lack of appropriate technologies to address these questions, the current paradigm states that the majority of membrane-bound Mtb resides in an early ‘arrested’ phagosome in macrophages.

With the advent of new technologies, data using live cell imaging show that in macrophages, the majority of individual Mtb phagosomes follow a conventional maturation pathway interacting with lysosomes (Schnettger *et al*. 2017). These results are in agreement with previous observations showing that Mtb can eventually survive upon trafficking into late endosomal compartments, either through Fc receptor-mediated phagocytosis or by co-infection with the pathogen Coxiella burnetti (Armstrong and Hart 1975; Gomes *et al*. 1999).

A kinetic study showed that Rab7 is transiently recruited to Mtb phagosomes, suggesting that dissociation of Rab7 from the Mtb phagosome is important for altered phagolysosome biogenesis (Seto *et al*. 2009; Seto, Tsujimura and Koide 2011). The presence of late endosomal markers in Mtb phagosomes was confirmed by using a novel method of phagosome purification using magnetic particles (Steinhauser *et al*. 2013).

Mtb can also be localised in spacious phagosomes, as originally described in EM studies (De Chastellier and Thilo 1997). The occurrence of tight and spacious Mtb phagosomes are conserved across host species and likely to be relevant *in vivo* since these spacious phagosomes have been observed in monocytes of TB patients (Russell, Mwandumba and Rhoades 2002), as well as in infected mice (Moreira *et al*. 1997), zebrafish (Hosseini *et al*. 2014) and *Dyctiostelium* (Barisch *et al*. 2015). Interferon-gamma (IFN-γ) is a key mediator of macrophage activation and resistance to intracellular pathogens. This cytokine increases interactions of Mtb phagosomes with the endosomal network, targeting mycobacteria to spacious, proteolytic compartments that reduce Mtb replication. Furthermore, this targeting of Mtb to spacious phagosomes also reduced phagosomal damage and access of mycobacteria to the cytosol. Without IFN-γ activation, fully virulent Mtb is able to bypass this targeting to spacious compartments, suggesting a complex interplay between immune activation and Mtb effectors (Schnettger *et al*. 2017).

### In a damaged compartment: causes and consequences

In addition to the bacterial Sec and Tat secretion pathways, Mtb possesses the ESX secretion system, a Type VII secretion system (T7SS) which is found to be non-essential for growth *in vitro*, but is required for bacterial virulence *in vivo* (Hsu *et al*. 2003). The first and most studied member of the five ESX secretion systems, ESX-1 is encoded by the region of difference (RD) 1 and its deletion results in attenuation of Mtb. Several Mtb proteins are secreted but the ESX-1 system and the 6-kDa early secreted antigenic target (ESAT-6; EsxA) and Culture filtrate protein 10 (CFP-10; EsxB) are the most abundant proteins found in supernatants from Mtb cultures.

Studies using a Mtb mutant lacking the RD1 region suggested that the ESX-1 system is implicated in the Mtb access to the cytosol, arguing for a role of ESX-1 in phagosomal membrane damage (Fig. 2) (van der Wel *et al*. 2007). There is also compelling evidence that specific cell wall lipids can contribute to membrane damage and the virulence-associated Phthiocerol dimycocerosates were shown in several systems to facilitate cytosolic localisation of Mtb in host cells (Augenstreich *et al*. 2017; Barczak *et al*. 2017; Quigley *et al*. 2017; Lerner *et al*. 2018). It is still not clear precisely how Mtb damages membranes; for example, we do not completely understand the complete structure of the assembled T7SS ESX-1 and how stable damaged phagosomes are, since damaged membranes are very unstable in a cytosolic (hydrophilic) environment. Moreover, the role of EsxA as pore toxin that was implicated in membrane damage is still unclear. The ability of EsxA to lyse membranes could be due to the way the protein is purified in the laboratory, which could explain the differences in results between groups (Refai *et al*. 2015). The purification method used is very important to the conformational state of EsxA which affects its ability to create pores. In agreement with this notion, in *M. marinum* some of the EsxA lytic activity was found to be due to detergent contamination (Conrad *et al*. 2017).

The fate of damaged Mtb phagosomes is relatively well characterised. Damaged phagosomes are now known to be rapidly recognised by the selective autophagy machinery (see below). Autophagy is a major intracellular pathway for the degradation and recycling of long-lived proteins and cytoplasmic organelles. Autophagy of foreign entities such as bacteria, viruses and parasites is termed xenophagy, an evolutionarily conserved mechanism classically observed to target and remove pathogens after host cellular invasion (reviewed in (Kimmey and Stallings 2016). After damage of Mtb phagosomes, Mtb components (e.g. DNA/RNA) access the cytosol, triggering signalling important for the innate immune response. During Mtb infection of macrophages, activation of cytosolic surveillance pathways is dependent on ESX-1, leading to type I interferon (IFN) and interleukin-1β (IL-1β) production. The inflammasome regulates IL-1β secretion and the nucleotidyltransferase Cyclic GMP-AMP synthase (cGAS), that induces synthesis of type I IFN via the cyclic di-nucleotide cGAMP in response to cytosolic DNA derived from viruses or retroviruses such as the human immunodeficiency virus (Cai, Chiu and Chen 2014). In this context, three independent reports showed the association of cGAS with antimycobacterial immunity (Collins *et al*. 2015; Wassermann *et al*. 2015; Watson *et al*. 2015). Altogether, these studies support a model whereby subsequent to Mtb infection, cGAS and mycobacterial DNA co-localize in the macrophage cytoplasm and form aggregates. This interaction initiates the synthesis of cGAMP by cGAS and consequent activation of the STING-TBK-1-IRF-3 signalling axis that drives transcription of IFN-β (Majlessi and Brosch 2015). STING is a signalling molecule essential for controlling the transcription of numerous host defence genes, including type I IFNs and pro-inflammatory cytokines, following the recognition of aberrant DNA species or cyclic dinucleotides in the cytosol of the cell (Barber 2015). These studies revealed the detection of cytosolic mycobacterial DNA by cGAS, emphasizing the concept of cytosolic access by Mtb. However, the *in vivo* relevance remains to be defined, since mice lacking cGAS are only moderately more susceptible to Mtb infection than their wild type littermates. Mycobacterial RNA accesses the cytosol through a SecA2- and ESX-1–dependent mechanism and activates the retinoic acid–inducible gene (RIG-I)/mitochondrial antiviral signalling protein (MAVS)/tank-binding kinase 1 (TBK1)/IRF7 signalling pathway. Activation of this RNA sensing pathway requires prior STING activation and works synergistically with the DNA sensing pathway to stimulate IFN-β production in host cells during Mtb infection (Cheng and Schorey 2018).

If cytosolic access occurs after complete disassembly of the phagosomal membrane, as observed in previous EM studies (Leake, Myrvik and Wright 1984; Myrvik, Leake and Wright 1984; McDonough, Kress and Bloom 1993) or it represents a dynamic process associated with membrane rupture and subsequent membrane repair, remains poorly characterised. Such conclusions require time-resolved data at ultrastructural resolution that are not readily obtainable (Simeone *et al*. 2016). Studies using a Fluorescent Resonance Energy Transfer (FRET)-based assay, were able to confirm that Mtb indeed accesses the cytosol in an RD1 dependent manner. (Simeone *et al*. 2012; Simeone *et al*. 2015; Wang *et al*. 2015) These reports showed that during infection of THP-1 macrophages, Mtb and BCG expressing ESX-1 displayed phagosomal rupture 3 to 4 days post-infection, which was followed by host cell death (Simeone *et al*. 2012). A later adaptation of the method allowed to observe a phagosome-to-cytosol connection *in vivo* in Mtb-infected phagocyte populations inside the lung parenchyma, granuloma and spleen of mice at the chronic phase of infection (Simeone *et al*. 2015; Simeone *et al*. 2016). This latter assay has been useful to define access of luminal components to the cytosol; however, it does not allow one to rigorously define if the bacteria are free in the cytosol (Fig. 2).

An important consequence of Mtb phagosome damage is the leakage of molecules from the phagosomal lumen. For example, biochemical properties that rely on an intact membrane such as pH will not be retained in a damaged phagosome. If damaged phagosomes are stable organelles, as shown for lysosomes (Skowyra *et al*. 2018), and displaying limited damage with pores of around 100 nm, remain to be investigated. The situation is similar for ions, cations and many amino acids that are contained within the lumen of a phagosome. It is likely that phagosomes that have been considered in several studies ‘lysotracker negative’, and therefore non-acidic, in fact are leaky phagosomes unable to maintain a gradient through the phagosomal membrane (Schnettger *et al*. 2017). It is becoming clearer that the host cell attempts to repair damaged Mtb phagosomes. One of the mechanisms is mediated by fusion of endosomal membranes with Mtb phagosomes that restore phagosomal membrane integrity (Schnettger *et al*. 2017). This pathway repairs damaged phagosomes, leading to the retention of Mtb in intact phagosomes that restrict Mtb replication *in vitro* (Schnettger *et al*. 2017). Interestingly, the endolysosomal repair machinery ESCRT-III is recruited into Mtb phagosomes in an EsxA dependent manner, suggesting a dynamic balance between bacterial damage and host repairing mechanisms (Mittal *et al*. 2018). During infection of *Dictyostelium discoideum* with *M. marinum*, the ESCRT-I component Tsg101, the ESCRT-III protein Snf7/Chmp4/Vps32 and the AAA-ATPase Vps4 are recruited after mycobacteria phagosome damage. While membrane damage induced by the ESX-1 secretion system of *M. marinum* are targeted by both ESCRT and autophagy, in absence of Tsg101, *M. marinum* accesses prematurely the cytosol, where the autophagy machinery restricts its growth (Lopez-Jimenez *et al*. 2018).

Notably, not all bacteria are able to induce damage and only a minor fraction is found to be ubiquitinated or positive for galectins, beta-galactoside binding lectins containing homologous carbohydrate recognition domains that recognise damaged membranes. These subpopulations of Mtb positive for galectins could result from dynamic changes between membrane-bound and cytosolic states at specific time points and dynamic studies (e.g. by using live-cell imaging) may reveal that a greater proportion become positive for galectins at a specific time point (Fig. 2). These dynamic cycles of damage and repair could depend on the timing and levels of the production of Mtb damaging factors. One possibility is that phenotypic variation within the bacterial population drives the heterogeneous response (Stanley and Cox 2013). For instance, stochastic fluctuations in ESX-1 activity may give rise to cell-to-cell variations in the flux of EsxA and thus create variations in membrane permeability from phagosome-to-phagosome (Ohol *et al*. 2010).

### In the cytosol

The emerging concept is that after Mtb-induced membrane damage occurs, there is communication or access to the cytosol and eventually the host cell will try to repair the damage via different mechanisms (Fig. 2). If this process is not properly contained, phagosome membrane disruption allows Mtb to freely localise in the cytosol. Crucially, the access of Mtb to the cytosol is critical for downstream signalling and modulation of the immune response. However, exactly how Mtb enters the cytosol, for how long it remains there and how the host cell recognises cytosolic Mtb remains to be fully characterised.

The cytosolic localisation of Mtb was reported in the early 80’s and subsequent reports using EM contributed to the idea that some Mtb could be free in the cytosol (Leake, Myrvik and Wright 1984; Myrvik, Leake and Wright 1984; McDonough, Kress and Bloom 1993). However, it was not clear to what extent this phenomenon was occurring. The main challenge to interpreting these data is related to the fact these studies were performed with conventional chemically fixed and plastic embedded cells, a method that is prone to artefacts. Methods and reagents available (e.g. antibodies) to investigate the phagosomal stage of Mtb were much better established and most of the research focused on this intracellular aspect of Mtb. However, studies using *M. marinum* (Mm) revealed that this close related of Mtb was free in the cytosol of macrophages. The cytosolic localisation of Mm was rapidly accepted because of the presence of actin tails in EM studies clearly confirmed Mm was free in the cytosol and propelled by actin tails (Stamm *et al*. 2003).

More than 20 years after the first descriptions of Mtb free in the cytosol, one study investigated again this aspect of the Mtb biology using a different approach. The use of thawed cryosections combined with immunogold EM allowed for good preservation of cellular membranes and to define cysosolic vs. phagosomal Mtb (van der Wel *et al*. 2007). However, as with earlier studies, this method used chemical fixation and the lack of a marker could not always mean absence of a surrounding membrane. By using both the absence of a membrane and a late endosomal marker, it was shown that the ESX-1 T7SS is one of the main Mtb factors responsible for the localisation of bacteria in the cytosol (van der Wel *et al*. 2007).

Despite several studies showing evidence that Mtb accesses the cytosol of host cells, the cytosolic localisation of Mtb is still debated and not widely accepted. For example, if the sub-cellular localisation of Mtb within cells and the physiological relevance of the cytosolic localisation is relevant for the disease is still disputed (Harriff, Purdy and Lewinsohn 2012). It is now clear that these original observations were, at least in part, correct based on more recent and compelling data from several independent groups using alternative methods. Moreover, the *in vivo* evidence of Mtb cytosolic access remains to be defined. A previous study carried out in mice using the FRET assay described above combined with flow-cytometry, showed that Mtb induces phagosomal rupture in the mouse model of infection. The assay is, as the one described for *in vitro* studies above, based on FRET changes depending on the β-lactamase activity present on the surface of bacteria. Although, the authors showed that this response is abrogated when mice are infected with Mtb strains lacking the ESX-1 secretion system, the possibility that some of these effects may have been caused by bacterial products translocating through permeable phagosomal membranes cannot be excluded (Simeone *et al*. 2015).

The current evidence argues strongly for a scenario whereby Mtb can be localised in functionally different intracellular locations, namely: (1) membrane-bound compartments, (2) transiently in damaged phagosomes or (3) free in the cytosol (Fig. 2). This mixed localisation has important implications for future interpretation of experimental data and also in drug development. It is clear that bacteria that is in a membrane bound compartment will be more affected by innate immune pathways that operate in the lumen of phagosomes (Reactive nitrogen species, ROS, hydrolytic enzymes of any class). The dynamics, functions and relevance of these sub-populations of Mtb, and in particular 2 and 3, in human macrophages remain unknown. In this context, if cytosolic Mtb constitutes a major target from a therapeutic standpoint remains to be defined (Russell 2016).

### In time

**Figure 3.**
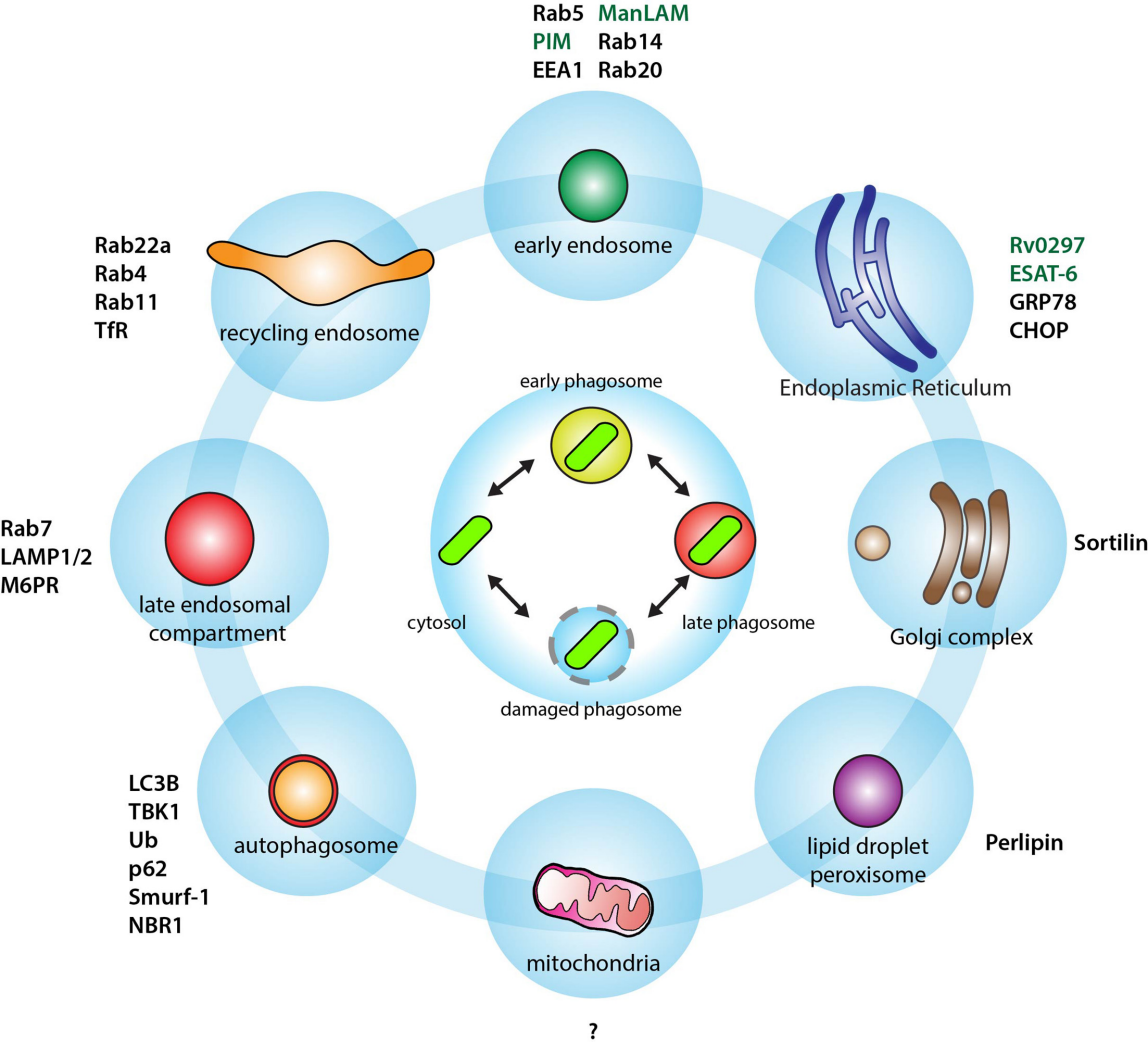
The time: spatiotemporal dynamics of Mtb interactions with host cell organelles. At least four different populations of intracellular Mtb (early phagosome, late phagosome, damaged phagosome and free in the cytosol) are localised in different environments and will interact dynamically with host cell organelles such as vesicles from the endocytic pathway (e.g. early, recycling and late endosomes); the autophagy pathway, endoplasmic reticulum, post-Golgi vesicles, the extensive network of mitochondria, lipid droplets and peroxisomes. Because of the dynamic nature of these Mtb populations, their interactions with host organelles will be different and likely regulated in a spatiotemporal manne by the bacteria and the host cell. Distinct molecular players involved in these interactions are shown. Known host factors are shown in black and Mtb factors in green.

The recently formed Mtb phagosome (e.g. after phagosomal membrane closure) will dynamically interact with numerous organelles derived from the endocytic, secretory and autophagic pathways (Fig. 3). Mtb phagosomes also interact with other membrane-less organelles such as lipid droplets (LD), double membrane organelles such as mitochondria and the multi-membrane network of the endoplasmic reticulum, although these interactions are poorly understood (Fig. 3). Crucially, these highly dynamic interactions between the endocytic and phagocytic pathway endorse the phagosome with antibacterial properties as part of the innate immune response.

### Interactions with the endocytic pathways

Pathogenic mycobacteria have evolved mechanisms to interfere with both (glyco)lipid and protein-mediated mechanisms that regulate the trafficking of bacteria to lysosomal organelles for destruction (Pieters 2008). Mtb glycolipids can interfere with phagosome-lysosome fusion through blocking a normal host trafficking event that is regulated by PI3P. PI3P is a host membrane component that is essential for phagolysosome biosynthesis (Roth 2004). Locally generated by PI3K on early endosomal and phagosomal membranes, PI3P represents a docking site for several regulatory proteins involved in the maturation of phagosomes into lysosomes, such as the hepatocyte growth factor-regulated tyrosine kinase substrate (Hrs) and EEA1 (Birkeland and Stenmark 2004; Pizarro-Cerda and Cossart 2004). The generation of PI3P regulates the delivery of phagocytosed cargo to lysosomes and Mtb interferes with this trafficking event by actively preventing PI3P accumulation on phagosomal membranes (Fratti *et al*. 2003).

Mannose-capped lipoarabinomannan (ManLAM), a macroamphiphilic lipoglycan exposed at the surface of Mtb cell envelope (Pitarque *et al*. 2008), is a key lipid that modulate phagocyte functions (Briken *et al*. 2004; Fukuda *et al*. 2013; Vergne, Gilleron and Nigou 2014). ManLAM interferes with the endocytic pathway by modulating calcium signalling and recruitment to the phagosome of EEA1, a tethering protein and Rab5 effector (Vergne *et al*. 2004a). EEA1 recruitment is necessary for delivering hydrolases such as Cathepsin D and vATPase from the Trans-Golgi-Network to the phagosome (Fratti *et al*. 2003). The impairment of phagosome maturation by ManLAM has been shown in different systems by several groups (Hmama *et al*. 2004; Kang *et al*. 2005; Welin *et al*. 2008).

Phagocytosis of dead but not live Mtb triggers an increase of cytosolic Ca^2+^ that results in activation of calmodulin-dependent kinase II (CaMKII) (Malik, Denning and Kusner 2000; Malik, Iyer and Kusner 2001). Inhibition of Ca^2+^, Calmodulin (CaM) and CaMKII prevents phagosomes containing dead Mtb from fusing with lysosomes. ManLAM prevents cytosolic Ca^2+^ concentration increase and through p38 MAPK activation, it contributes to privation of PI3K on the Mtb containing phagosomes and reduction of Rab5 levels in early endosomes, thus providing a rationale for its effect on limiting EEA1 recruitment and phagosome maturation (Fratti *et al*. 2003; Vergne, Chua and Deretic 2003; Vergne *et al*. 2004b).

There is also evidence that Mtb phagosomes interacts with Golgi-derived vesicles that contain enzymes implicated in innate immunity (Fig. 3). Sorting of luminal and membrane proteins into phagosomes is critical for the immune function of this organelle. Sortilin, also known as neurotensin receptor 3 is a transmembrane receptor that transports lysosomal proteins from the trans-Golgi network into lysosomes, as an alternative route to mannose-6-phosphate receptors (Braulke and Bonifacino 2009). The phagosomal association of sortilin is critical for the delivery of acid sphingomyelinase and required for efficient phagosome maturation. Furthermore, *in vitro* and *in vivo* studies showed that sortilin is implicated in a Golgi-to-phagosome pathway that is required for controlling intracellular mycobacteria and lung inflammation (Vazquez *et al*. 2016).

### Interactions with the autophagic pathway

Whereas there is a large body of literature linking xenophagy as an anti-mycobacterial pathway (Deretic ; Gutierrez *et al*. 2004), how and when Mtb is targeted to autophagic compartments, and eventually autophagolysosomes in human macrophages is still unknown. There are at least four possible alternative mechanisms: (1) direct sequestering of intact Mtb phagosomes by phagophores, the membranes that initiate autophagy; (2) targeting via selective autophagy of cytosolic bacteria and/or damaged phagosomes; (3) direct fusion of autophagosomes with intact phagosomes and (4) direct recruitment of autophagic proteins (e.g. LC3B) into Mtb phagosomes, as in non-canonical autophagy (Fig. 3). This information is critical, given the data showing that Mtb can eventually block the fusion of autophagosomes with lysosomes (Romagnoli *et al*. 2012; Lerner *et al*. 2016) and data showing that Mtb can evade the autophagic responses in the mouse model of TB (Kimmey and Stallings 2016). Induction of autophagy promotes maturation and acidification of Mtb phagosomes and their conversion into mycobactericidal organelles (Gutierrez *et al*. 2004; Harris *et al*. 2007; Fabri *et al*. 2011) through the canonical autophagy machinery or alternative pathways such as non-canonical autophagy (Cemma and Brumell 2012).

Compelling evidence shows that Mtb is targeted to autophagosomes via ubiquitination. However, it is not yet clear if the substrates for ubiquitination in infected cells are intact mycobacteria that escaped into the cytosol, mycobacterial components inserted on host membranes, secreted bacterial molecules, damaged host membranes or simply host cell proteins found on Mtb phagosomes. Independently of the substrate identity, a small fraction of damaged Mtb phagosomes are tagged with ubiquitin, and ubiquitin moieties are recognised by autophagic adaptors and the selective autophagic machinery that will target Mtb into autophagosomes, and presumably into the lysosomal degradation pathway (Watson, Manzanillo and Cox 2012). Evidence argues that different Mtb clinical strains may be differentially recognised by the selective autophagy pathway, suggesting different behaviours of different Mtb isolates in their ability to damage host membranes (Kumar *et al*. 2010; Haque *et al*. 2015).

A more detailed analysis of autophagy factors such as Sequestosome 1/p62 has established that the entire pathway is important for antimycobacterial action of autophagy (Ponpuak *et al*. 2010). A specific manifestation of this is that initiation of autophagy generates and delivers a mixture of antimicrobial peptides, known as cryptides, into Mtb phagosomes (Ponpuak and Deretic 2011). These peptides are produced through autophagic proteolysis of otherwise innocuous cytosolic proteins such as ribosomal proteins (Ponpuak *et al*. 2010) and ubiquitin (Alonso *et al*. 2007). As already mentioned, a fraction of intracellular Mtb bacilli escape from phagosomes into the cytoplasm or are in contact with the cytosol (van der Wel *et al*. 2007). The bacilli that are in the cytosol represent a minor proportion of the total intracellular Mtb, but are nevertheless subject to targeting through selective autophagy (Watson, Manzanillo and Cox 2012).

The kinase TBK-1 also plays a critical role in the regulation of mycobacterial control by autophagy (Pilli *et al*. 2012). TBK-1 is required for IL-1β-induced clearance of Mtb by autophagy since the TBK-1 inhibitor BX795 or TBK-1 depletion reduces mycobacterial killing when autophagy is induced by IL-1β (Pilli *et al*. 2012).

The elimination of Mtb via an ubiquitin-dependent mechanism where phagosomes enclosing bacteria are tagged with ubiquitin chains is partially regulated by the E3 ligase Parkin that triggers K63-ubiquitination of mycobacteria. Macrophages lacking Park2 show a significant reduction in ubiquitin-positive mycobacteria compared with normal cells (Manzanillo *et al*. 2013). Infected Park2^−/−^macrophages also revealed decreased recruitment of the ubiquitin adaptors, P62, NDP52 and NBR1, and the autophagy proteins, LC3 and ATG12, to mycobacterial cells, compromising their efficient elimination (Manzanillo *et al*. 2013). Ubiquitin tagging of Mtb is likely more complex since other E3 Ubiquitin ligases have been implicated in Xenophagy of Mtb (Franco *et al*. 2017; Pei *et al*. 2017).

On the other hand, Mtb has been shown to subvert the function of some intrinsic host mechanisms favouring their own survival in host cells. For instance, Coronin 1a (CORO1A) is a host F-actin-binding protein that is activated by Mtb and impairs autophagosome formation around the bacillus-containing phagosomes. This observed reduction on autophagosome formation is likely the result of impaired activation of the p38 MAPK necessary for autophagy induction through TLR signalling (Seto, Tsujimura and Koide 2012). In addition, the Mtb protein Eis has been shown to acetylate Lys-55 in a JNK-specific phosphatase (Kim *et al*. 2012). JNK action is important for activation of the autophagy regulator Beclin-1, and thus Eis may modulate autophagy (Shin *et al*. 2010; Ganaie *et al*. 2011; Kim *et al*. 2012). A mycobacterial glycolipid lipoarabinomannan has also been reported as a strong inhibitor of autophagy (Shui *et al*. 2011). The Mtb effector protein EsxA is secreted via the ESX-1 secretion system and blocks Mtb phagosomal maturation into degradative autolysosomal organelles (Romagnoli *et al*. 2012; Zhang *et al*. 2012). Nonetheless, the precise mechanisms and pathways used by Mtb to evade autophagy are poorly understood (Deretic 2014).

One of the non-canonical pathways of autophagy is LC3-associated phagocytosis (LAP) where LC3 is rapidly conjugated to a single-membrane phagosome in a process that is independent of the autophagy initiation complex Ulk-1 (Martinez *et al*. 2015). LAP is initiated by signalling through pathogen recognition receptors such as TLR2 and CLRs, resulting in the recruitment of the nicotinamide adenine dinucleotide phosphate oxidase (NADPH oxidase) to the phagosome. The NADPH oxidase generates Reactive Oxygen Species, which are essential for LAP. In addition, Rubicon, a negative regulator of the PI3K complex, which is also required for LAP, stabilizes the NADPH oxidase. Although little is known about the role of LAP during Mtb infection, there are data showing that, similar to canonical autophagy, Mtb inhibits LAP through CpsA, a LytR-CpsA-Psr (LCP) domain-containing protein which disrupts the NADPH oxidase. However, how CpsA impairs NADPH oxidase recruitment and eventually LAP remains to be investigated (Köster *et al*. 2017).

### Interactions with other organelles

It has been postulated that interactions between Mtb and LD are important for the infection. Foamy macrophages accumulate in granulomas during mycobacterial infections, a process that Mtb mycolic acids can induce (Peyron *et al*. 2008). Moreover, these investigators found that mycobacteria were ultimately delivered into the LD, where bacilli accumulate lipids (Peyron *et al*. 2008). A later study showed that accumulation of lipids by Mtb within foamy macrophages mainly results from the incorporation of fatty acids derived from host TAG in a process largely mediated by mycobacterial triacylglycerol synthase 1 (Daniel *et al*. 2011). These observations indicate that Mtb can use host LD as a source of nutrients. However, there are limited dynamic studies on how LD associate with Mtb during intracellular infection, particularly in human host cells, in real time. In addition, most of the original observations lack quantitative analysis at the single cell level (Fig. 3). In contrast, there is another study suggesting that LD formation is not a Mtb-driven process, but rather occurs as a result of IFN-γ activation of macrophages and as part of a host defense mechanism (Knight *et al*. 2018).

Interaction of mycobacteria with the host macrophage also results in plasma membrane microdisruptions (Roy *et al*. 2004; Divangahi *et al*. 2009). Resealing of these lesions, a process crucial for preventing necrosis and promoting apoptosis, required translocation of lysosomal and Golgi apparatus-derived vesicles to the plasma membrane. Plasma membrane repair depended on prostaglandin E2, which regulates synaptotagmin 7 (Syt-7), a calcium sensor involved in the lysosome-mediated repair mechanism (Divangahi *et al*. 2009). Although there is insufficient evidence supporting a role of the ER during Mtb infection, previous reports have shown an association between mycobacterial secreted proteins and the activation of the ER stress response, which could mediate apoptosis of macrophages and epithelial cells during infection (Choi *et al*. 2010; Grover *et al*. 2018).

Bacterial effectors can target mitochondria and manipulate their function and dynamics (Jain, Luo and Blanke 2011; Lum and Morona 2014; Lobet, Letesson and Arnould 2015; Chowdhury *et al*. 2017; Escoll *et al*. 2017) (Fig. 3). The effects of mycobacterial proteins and the interaction of Mtb with the mitochondrial network remain largely unexplored. Recently, the host Immune-Responsive Gene 1 (Irg1; also called Acod1), a mitochondrial enzyme induced under inflammatory conditions that produces the metabolite itaconate, has been shown to be regulated Mtb. By using Irg1^−/−^and Irg1^fl/fl^ conditional gene-deleted mice this study showed that complete absence of Irg1 during Mtb infection resulted in severe pulmonary disease and ultimately death (Nair *et al*. 2018). Although macrophage necroptosis, a programmed form of necrosis that is dependent on activation of receptor-interacting kinase 3 (RIPK3 and the mixed lineage kinase domain-like pseudokinase), is associated with depolarized mitochondria and impaired ATP synthesis, known hallmarks of Mtb-induced cell death, the effects of mycobacterial proteins and the interaction of Mtb with the mitochondrial network remain largely unexplored.

### Challenges to study human macrophage-Mtb interactions *in vitro*

#### Human macrophages

**Figure 4.**
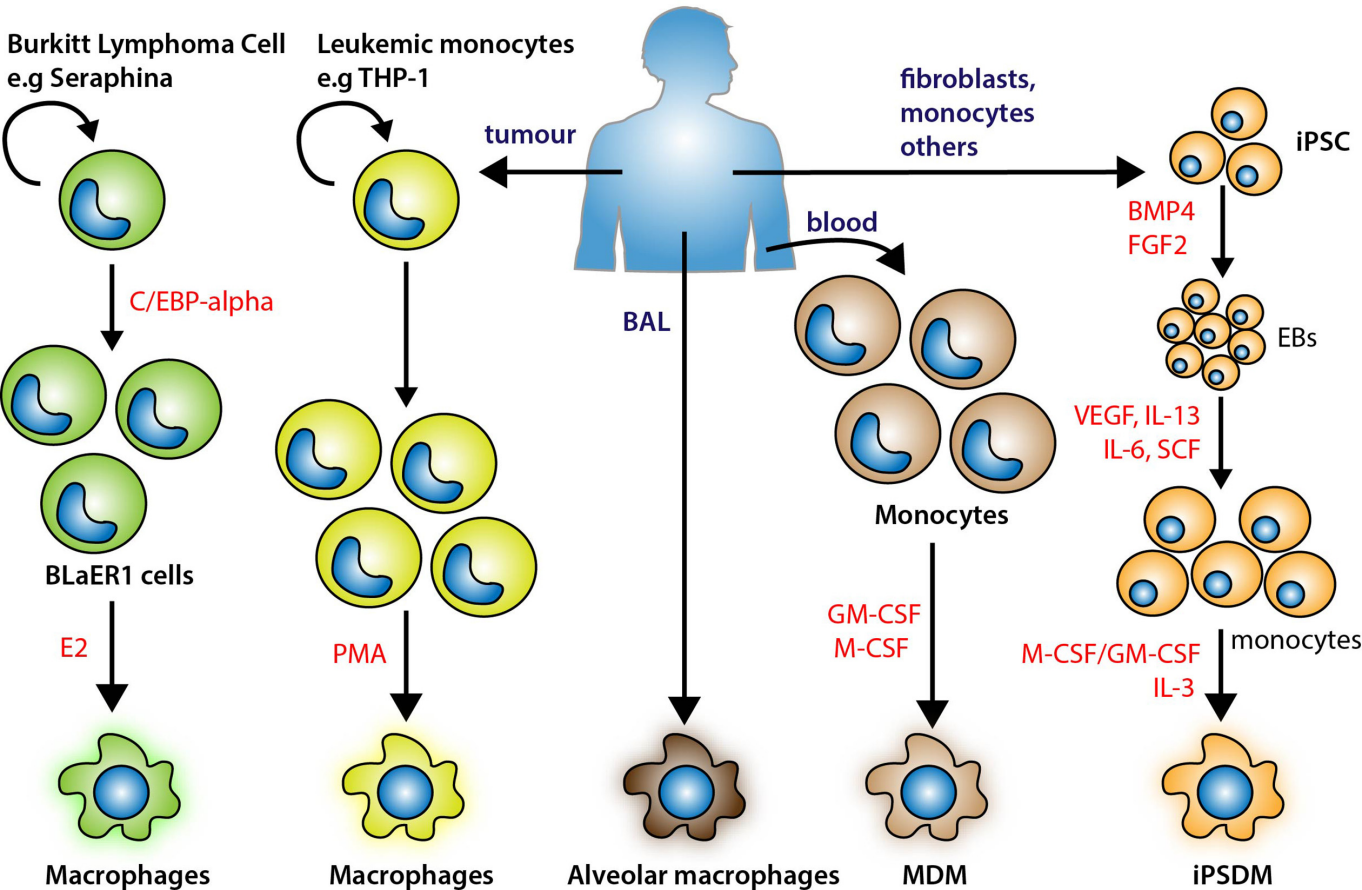
Human macrophage systems to study Mtb-macrophage interactions *in vitro*. A commonly used human macrophage source is derived from monocytic leukaemia cell lines, like THP-1 and U937 cells that require a ‘differentiation’ step typically achieved with phorbol esters and/or Vitamin D treatment. An alternative source is based on lineage conversion from malignant B-lineage cells to monocytes/macrophages that is caused by the inducible nuclear translocation of a C/EBPα transgene, BLaER1 cells. Although trans-differentiated BLaER1-monocytes transcriptome is highly similar to that of their primary monocytes, if this method can recapitulate the complete functional properties of human primary macrophages requires further validation. Alveolar macrophages can be obtained in limited numbers from broncholavelolar lavage or lung biopsies. Another widely used method is the isolation of monocytes from peripheral blood that are subsequently differentiated using recombinant differentiation factors such as macrophage colony stimulating factor (M-CSF) or granulocyte macrophage colony stimulating factor (GM-CSF). With advances in our understanding of stem cell biology, the use of iPSDM. It involves a stepwise differentiation programme that includes haemogenic endothelial specification achieved and maintained via the introduction of bone morphogenetic protein 4(BMP4), fibroblast growth factor 2 (FGF2) and vascular endothelial growth factor (VEGF). Further differentiation requires signalling from the activin–Nodal and WNT pathways that is achieved through endogenous signalling via the formation of embryoid bodies (EBs). When EBs form, they are transferred to an adherent surface and exposed to cytokines, such as M-CSF and IL-3 to promote the generation of monocytes, which will be finally differentiated into macrophages.

Although Mtb is a human pathogen, the majority of the *in vitro* host-pathogen studies in TB have been carried out in mouse cells. Detailed cell biology studies of Mtb infected human macrophages in the literature are limited since, until recently the available *in vitro* systems were not ideal. The most commonly used human macrophage models are cancer cell lines such as the human monocyte-like cell line THP-1 (Tsuchiya *et al*. 1980) or U937 (Sundstrom and Nilsson 1976) (Fig. 4). However, these cells need to be chemically activated with phorbol esters before use and are karyotypically abnormal. Moreover, by definition, they are not terminally differentiated macrophages, complicating the interpretation of results. Many signalling pathways are partially active (or completely absent) in these cells, such as the inflammasome pathways (Gaidt *et al*. 2016). Immunity in these proliferating cells might be different from physiological conditions and this has to be considered when selecting these cell lines as models to study immune responses. Another macrophage cell line model is the BLaER1 cells that uses lineage conversion by the inducible nuclear translocation of a C/EBPα transgene from malignant B-lineage cells to monocytes/macrophages. Using this model, it has been possible to characterize a novel species-specific NLRP3 inflammasome pathway that existed in human and porcine peripheral blood mononuclear cells but was absent from murine peripheral blood mononuclear cells and THP-1 cells (Gaidt *et al*. 2018). A great advantage of the cell line-based macrophage systems is the possibility to perform genetic-based studies using CRISPR/Cas9 (Fig. 4).

It is thought that after infection in humans Mtb travels into the lungs and once there, alveolar macrophages will phagocytose the bacilli. There is little evidence in humans about this critical event, there are data in mouse models of TB indicating that alveolar macrophages are important for disease progression and dissemination (Cohen *et al*. 2018). Alveolar macrophages are therefore considered the niche for Mtb and to carry out experiments with primary alveolar macrophages clearly represents a more physiological setting. One of the caveats of this model are the cell numbers since these cells are obtained from bronchoscopy and bronchoalveolar lavage fluid or post-mortem lung tissue. Therefore, these cells are difficult to culture in sufficient numbers for extensive *in vitro* experiments (Fig. 4). There are however available protocols that are suitable for experiments (O'Leary *et al*. 2014) to investigate macrophage metabolism (Gleeson *et al*. 2018) and autophagy (Coleman *et al*. 2018).

A widely used cellular system of human macrophages is peripheral blood-derived mononuclear cell macrophages (MDM) that are differentiated *in vitro* using a wide range of cytokines. Human primary macrophages are physiologically relevant but highly variable across donors and are not amenable to genetic manipulation using CRISPR/Cas9 or other gene editing systems. In contrast to mouse macrophages, one important physiological feature of MDM is that despite the expression of inducible nitric oxide synthase (iNOS), these cells produce low (or absent) levels of nitric oxide after activation. It is important to consider that independently of the method used to isolate and differentiate the MDM, these cells have a limited lifetime that is normally about 2 weeks. Careful studies that compared oxygen levels, source of serum, and cytokines were able to significantly extend the survival of MDM for longer than 2 weeks (Vogt and Nathan 2011). Therefore, in well-defined systems MDMs represent a good source of physiologically relevant macrophages.

The fast-moving field of stem cell research has significantly increased the options to work with human macrophages (Lee, Kozaki and Ginhoux 2018). In particular, human induced pluripotent stem cell- (hiPSC) derived macrophages (iPSDM) represent a very attractive system to study the biology the human macrophages (van Wilgenburg *et al*. 2013) (Fig. 4) since gene expression studies showed that iPSDM resembles human monocyte-derived macrophages (Hale *et al*. 2015). One of the most useful advantages is the possibility to use CRISPR/Cas9-based genome editing. Moreover, there are continuous efforts in multiple laboratories to differentiate hiPSC into different physiological human alveolar-like macrophages, a system that would be extremely useful to study Mtb infection. Although how closely these macrophages represent physiologically relevant macrophages remains to be defined (Lee, Kozaki and Ginhoux 2018). These cells are derived from single donors (either individual donors or from a stem cell bank) and experiments need to be repeated with several donors. The possibility to use iPSC from patients with specific mutations to investigate biological functions is also very attractive. However, in addition to the costs associated with this method, an important aspect to consider is that these cells contain a specific genetic background with natural mutations. So, if mutations affecting the pathway under study are present, this should be analysed before starting with any particular clone in cases where the genome of that particular clone is available. Overall, iPSDM represent a powerful and genetically amenable tool to investigate the cell biology of human macrophages during infection and it will certainly be more used in future studies.

Another aspect to consider is that biology is in three dimensions. The relevance of 3-dimensional organisation in the context of Mtb infection and granuloma formation is key to our understanding of TB pathogenesis and 3-dimensional cellular systems have been developed for studying TB treatment (Bielecka and Elkington 2018). In these models, infected primary human cells are co-cultured with collagen matrix gels, agarose beads or agarose-coated plates and it could also include epithelial cells and fibroblasts (Fonseca *et al*. 2017). A microsphere system, has also been used to test efficacy of anti-TB drugs. In this system, microspheres are generated within a cell encapsulator, incorporating Mtb-infected primary human cells including monocytes and T cells within extracellular matrix (Tezera *et al*. 2017). Interestingly, lung organoids and lung-on-a-chip models have been investigated in the context of infection (Sachs *et al*. 2019), however, further development and validation is required, for example by incorporating different cell types relevant to the infection, before applying these experimental systems to the study of TB disease.

#### Localisation of Mtb within host cells

**Figure 5.**
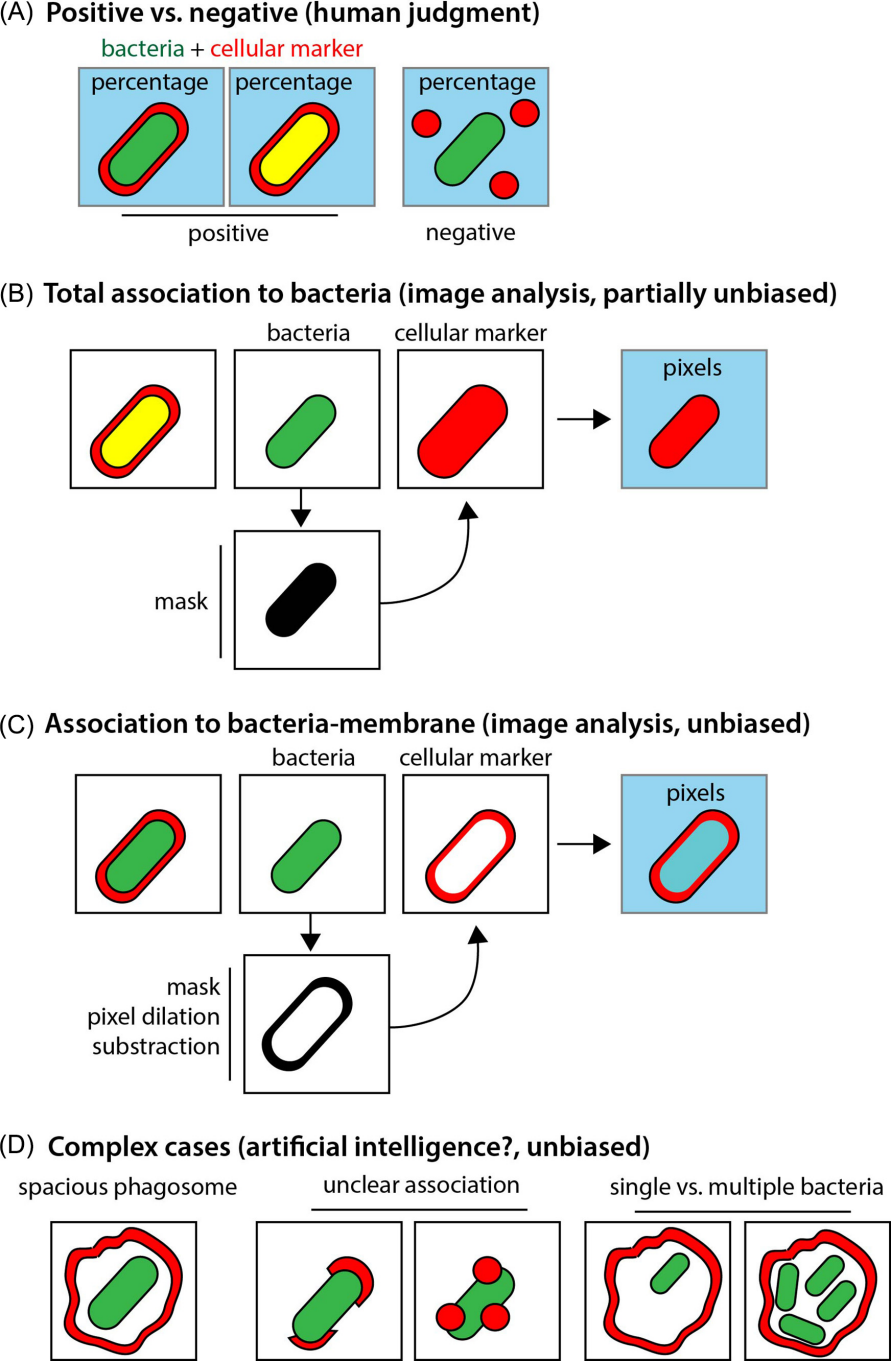
Different fluorescent- based approaches to study the intracellular localisation of Mtb. **A**, One of the most widely used strategies to study bacteria localisation is based on a biased judgement to determine if a particular intracellular marker (shown in red) co-localise with bacteria (shown in green). **B**, Alternatively, in cases where the cellular markers co-localise almost completely with bacteria, a more quantitative and unbiased method can be employed using a masking strategy to select bacteria and applying this selection to the cellular marker channel/image to determine the pixel number and intensity associated with bacteria. **C**, A variant of this unbiased method can also be applied when the intracellular marker is associated with the bacteria membrane but it does not cover all the surface. In this case, is necessary to dilate the original bacteria mask. The basic effect of this operator (on a binary image) is to gradually enlarge the boundaries of regions of foreground pixels. Then, the bacteria pixels will be subtracted and the resulting mask will be used to quantify the corresponding pixels in the cellular marker image/channel. **D**, Examples where unbiased methods are not suitable for quantification and positive or negative co-localisation is difficult to determine since the distribution of the intracellular marker is asymmetric or it only partially covers the bacteria surface. In these cases, artificial intelligence represents a promising methodological advance.

Determining the localisation of Mtb within human macrophages (and other host cells) is critical to understand how cellular pathways contribute to bacterial control or replication. Defining whether Mtb is in an acidic phagolysosome, an autophagosome or remains in an early endosome is one of the of the cornerstones of the cellular microbiology of Mtb (and other intracellular pathogens). Understanding where Mtb is localised within host cells is important to understand signalling pathways triggered by Mtb. The majority of these studies of Mtb-infected cells are carried out by measuring the percentage of colocalisation of a selected cellular marker with Mtb. It is common practise to use a potentially biased quantification strategy that utilises human judgement to determine a positive or negative association of bacteria to the marker. This method, that consider events in the order of hundreds, is problematic because the localisation of Mtb is highly heterogeneous within a single cell, it shows a remarkable variability from cell to cell and it is difficult to define when an intracellular Mtb is positive or negative (Fig. 5). Despite its inherent problems, this method is commonly used and represents a useful choice in difficult cases, for example when bacteria are localised in an asymmetric and large compartment and semi or quantitative methods are imprecise (Fig. 5).

In the last decade, there has been a considerable advance in single cell quantitative image analysis. The use of unbiased quantitative image-based methods, which facilitates measuring large numbers of mycobacteria (in the order of thousands) and their association with specific markers circumvents this problem. This type of studies has considerably increased our knowledge of Mtb localisation and revealed a remarkable heterogeneity of intracellular populations. One of the most commonly used ways to do it is by masking bacteria and analysing the fluorescence of the cellular marker associated to single bacteria (Fig. 5). This approach is sometimes not useful when analysing membrane markers surrounding bacteria. For that, another approach that uses several steps of image processing (Fig. 5) can accurately measure a ring around single ‘masked’ bacteria. There are however cases where it is still difficult to use an image-based unbiased quantitative analysis. For example, in cases where bacteria are in a large and asymmetric compartment and a membrane marker needs to be measured. There are other situations where the marker is not clearly associated but overlaps with bacteria, this can be challenging when analysing markers of damaged membranes and/or phagosomes, that are partially associated with the bacteria (Fig. 5). Finally, there are conditions where a compartment positive for a specific marker contains either single or multiple bacteria. In that case, additional steps are required to distinctively quantify these events and evaluate their functional relevance. In the last few years, machine intelligence algorithms started to be used in the field of host-pathogen interactions and provide a promising approach to analyse these complex cases (Fisch *et al*. 2019).

Ideally, fluorescent studies using fluorescent probes and antibody-based labelling should be complemented and correlated with EM studies to observe membranes at the ultrastructural level. CLEM is useful because both fluorescence- and EM-based information is combined but it is not suitable for high content studies. Tokuyasu thawed cryosectioning and immunolabelling is mostly the preferred method to visualise cellular markers and ultrastructure. This technique requires care and skill and in the case of Mtb, careful standardisation since most of the antibodies will cross-react with mycobacteria. EM stereology represents a very useful approach when analysing large populations of intracellular bacteria but in general, cellular markers are not considered. Given that the bacterial populations are very heterogeneous, correlation is one of the most desirable approaches to define localisation of intracellular Mtb.

#### Intracellular Mtb viability

**Figure 6.**
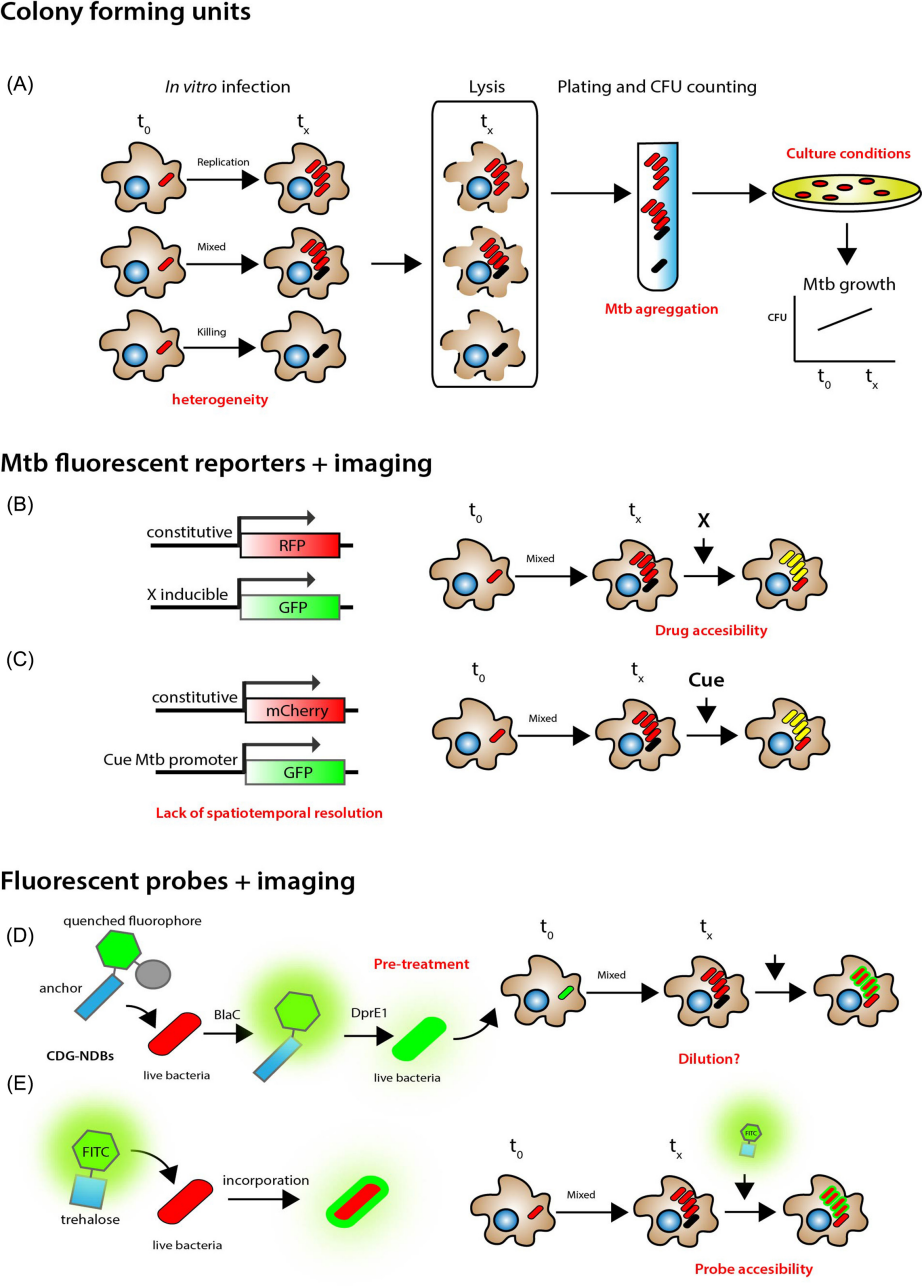
Measuring Mtb viability in host cells after infection. **A**, Currently, the conventional method to evaluate viability of Mtb relies on bacterial CFU enumeration on agar plates. This is a time-consuming approach due to the slow growth rate of Mtb that takes about 3–6 weeks to observe visible colonies on agar plates. One of its main disadvantages of this method is that clumps of bacteria cells can be miscounted as single colonies. **B**,The graph illustrates a genetic approach where Mtb constitutively express mCherry (red) and express GFP (green) using an inducible TetON promoter. After tetracycline induction ‘live’ bacteria express both GFP (green) and mCherry (red) fluorescence, while ‘dead’ bacteria are only mCherry positive (red). **C**, A similar strategy to (B) where the Mtb reporter strains are constructed to constitutively express mCherry (smyc′::mCherry) that enables the visualization of all bacteria combined with the expression of GFP that is regulated by promoters that respond to environmental cues (for example, pH). **D**, Dual-targeting fluorogenic probes (CDG-DNBs) containing a BlaC-sensing unit, a caged fluorescent reporter, and a DprE1-binding unit for signal trapping. CDG-DNBs pass the Mtb cell wall through porins, BlaC and DprE1 enzymes located in the peptidoglycan layer and at the outer membrane will react with the probes. Then, BlaC will hydrolyze the lactam ring to activate the fluorophore, and DprE1 will covalently bind the anchor unit for fluorescence immobilization. The combined actions of BlaC and DprE1 would enable fluorescent labelling of single Mtb. Bacteria without any BlaC and/or DprE1 activity would not fluorescently label due to the lack of fluorescence activation (no BlaC) or signal retention (no DprE1) in cells. **E**, A FITC-trehalose probe that exploits the processing by Mtb Ag85 enzymes is specifically incorporated into Mtb growing *in vitro* and within macrophages.

At present, researchers mostly rely on rates of replication or killing of the mycobacteria at the population level e.g. by Colony Forming Unit (CFU) determination. The method of CFU to evaluate viability of Mtb in cellular models of infection is the most widely used but unfortunately the most problematic too (Fig. 6). CFU has several limitations and represents at best an approximation of the bacterial numbers in a defined system (e.g. macrophages infected with Mtb). The first point to consider is that bacteria recovered on agar plates of commercially available microbiological media are the ones able to grow under these defined conditions. This led to the concept of viable but non-culturable bacteria and this phenomenon could interfere with the interpretation of results. Another confounding factor is that the CFU method is based on the idea that one colony on the plate represents one bacteria, it is likely that is not the case in Mtb with its well-known ability to clump and form aggregates in solution (Fig. 6). Combined with the high variability associated to this technique, prone to high errors when working in Biosafety Level 3 lab with very high volume of samples and the fact that colony counts do not correlate to the dilution factor; this method needs to be carefully considered. Even more problematic is the fact that in cell biology studies that investigate the intracellular localization of Mtb, there is in general little information about the viability status of the bacteria. The main obstacle in elucidating the precise fate of Mtb has been the difficulty in unequivocally establishing if a single Mtb localised in a defined compartment (e.g. a late endosome) is able to replicate, or not, or is killed. Even under conditions where the majority of bacteria are killed, the ability of a minor pool to replicate, or to remain alive in a non-replicating state can have major implications for the future course of the infection.

In the last years, to overcome the problems associated to the CFU assay and trying to obtain information about the physiological state of Mtb within cells, image-based approaches, mostly based on fluorescence, have been developed.

A ‘live/dead’ Mtb H37Rv reporter that constitutively expresses mCherry and conditionally (following anhydrotetracycline induction) expresses GFP in transcriptionally active bacteria has been developed (Martin *et al*. 2012). This system defines live bacteria as transcriptionally active and assumes the ‘inducer’, in this case tetracycline, is able to penetrate and reach all intracellular bacteria (Fig. 6). The ratio red/green (live/dead) pixels are averaged in untreated and antibiotic treated infected cell populations and plotted as % ‘live’ of control untreated infected cell populations. Live/Dead Mtb following treatment with the inducer. In this system where ‘live’ Mtb are mCherry + (red) and GFP + (green) while ‘dead’ Mtb are mCherry + the fluorescent ratio in cell populations is used to measure bacterial viability. Interestingly, using this approach it was found that after one day of rifampicin treatment, around 20% bacteria were still alive in Mtb-infected mouse macrophages (Martin *et al*. 2012).

A similar dual reporter strategy, that does not measure directly bacterial viability, has been used by combining promoters that respond to specific environmental cues in host cells and *in vivo*. In this system, one promoter drives constitutive expression of a fluorophore whereas a second promoter (that is regulated by environmental cues) controls the expression of a different fluorophore. Using this approach, it has been possible to define how Mtb respond to stress and nutrients in host cells and *in vivo* (Tan *et al*. 2013; Sukumar *et al*. 2014). Because it utilises highly stable GFP as a readout, with this system the spatiotemporal resolution is limited.

Another small molecular probe that uses a very elegant dual-targeting strategy has been developed to specifically label mycobacteria (Cheng *et al*. 2018). This probe discriminates between live and dead *M. bovis* BCG. The probe is named CDG-DNB3 and fluoresces upon activation of the β-lactamase BlaC (a hydrolase expressed in Mtb). In a second step, the fluorescent product is retained within the bacilli through covalent modification of the Mtb enzyme decaprenylphosphoryl-β-d-ribose 2'-epimerase (DprE1). The probe works when bacteria are pre-treated and used to infect mouse macrophages and could potentially work to monitor viability of Mtb within host cells (Fig. 6). In long term experiments needed for monitoring Mtb replication, the system could be potentially diluted over time. Overall, it is not trivial to discriminate between live and dead bacilli when looking at infected cells and it depends largely on how Mtb viability is defined. Most of the systems have some difficulties with the interpretation of the results and ideally, a combination of more than one method is required.

Other probes exploited specific sugars that are only present in mycobacteria. The disaccharide sugar trehalose is absent in mammals and synthesized by Mtb via the enzymes Ag85A, Ag85B and Ag85C. These enzymes are sufficiently promiscuous to process a variety of trehalose analogs that are fluorescently labelled and trehalose-probe analogs are also efficiently anchored to Mtb. Trehalose probes are selectively taken up by live cells and label live Mtb in infected mammalian cells. These probes were able to differentially label relevant populations of intracellular Mtb that reflects intracellular localisation (Backus *et al*. 2011). These probes have not been extensively used to label replicating Mtb in macrophages. Some additional trehalose probes have been developed to detect Mtb *in vitro* and sputum and could represent another useful probe to monitor intracellular replication of Mtb (Kamariza *et al*. 2018).

#### Intracellular Mtb replication

**Figure 7.**
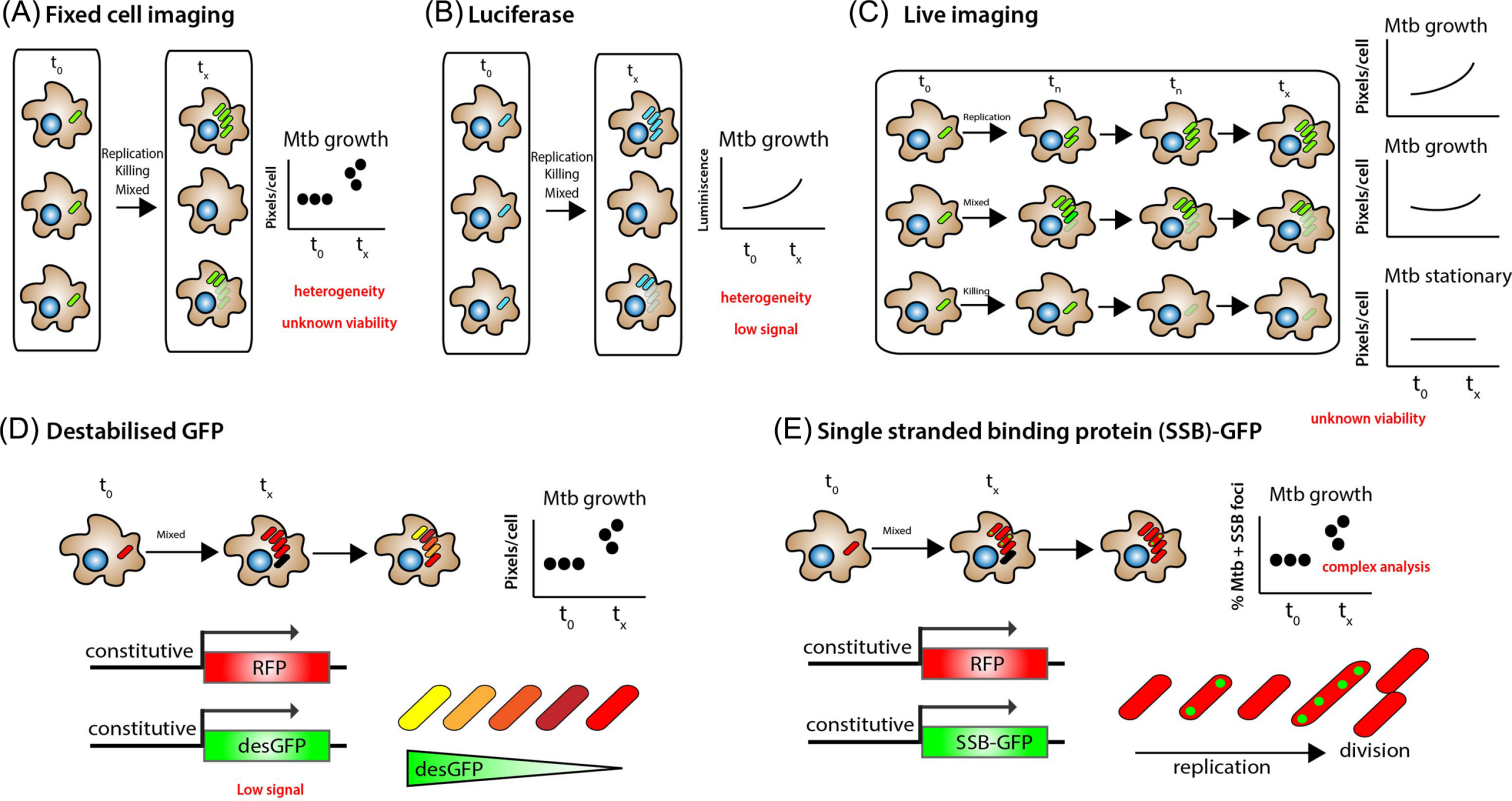
Measuring Mtb intracellular replication. **A**, One approach to follow Mtb replication involves the quantification of fluorescent Mtb strains through time at single cell level in fixed cells. Although this method can be adapted to high-content imaging, it does not take into account cellular heterogeneity and does not allow to fully distinguish live from dead bacteria. **B**, Shows the Luciferin-luciferase reporter. This system is useful for studying cell populations but does not allow for single-cell analysis. **C**, A more time-consuming approach that it does allow spatiotemporal resolution is to evaluate intracellular bacteria replication by live cell imaging. **D**, Shows genetically encoded fluorescent reporter in combination with quantitative time-lapse microscopy. It uses a destabilized variant of green fluorescent protein (GFPdes) with a plasmid expressing a stable red fluorescent protein (DsRed2) from a constitutive promoter, as internal control. In this example, stationary-phase populations exhibit a drop in GFPdes expression followed by the appearance of a subset of non-replicating bacteria displaying high levels of fluorescence. **E**, Another replication reporter uses a SSB that is fused to GFP, present on a replicating plasmid that contains a constitutively expressed mCherry. The SSB-GFP reporter defined cell cycle timing, with SSB-GFP foci present for periods of DNA replication and disappearing when the bacteria stop growing or it is during stationary phase.

In the last few years, many groups have developed fluorescence reporters in Mtb to monitor bacterial replication *in vitro*. Macrophages are a niche for Mtb and resting macrophages *in vitro* provide very good conditions for bacterial replication. Understanding the location where Mtb replicates but also the conditions that restrict growth is critical to further delineate the cellular immune response to Mtb. This aspect however has been less investigated and establishing if a single Mtb localised in a defined compartment is able to replicate has been difficult. To measure bacterial replication quantitatively and at the single-cell level in host cells has been challenging due to the slow replication of mycobacteria and the limited availability of live cell imaging systems in Biosafety Level 3. One way to follow intracellular replication of Mtb is by monitoring active growth of fluorescent Mtb through time at single cell level in fixed cells (Fig. 7). With these methodologies is however not possible to determine if bacteria are alive or dead as discussed above. Moreover, without spatiotemporal resolution, it is difficult to define whether Mtb was actually growing within the same cell due to cell death and efferocytosis.

Luciferin-luciferase systems are widely utilised in mycobacterial research (Andreu *et al*. 2010; Andreu *et al*. 2012). These reporters mainly exploits the firefly, *G. princeps* and Renilla Luciferase systems. In addition, bacterial luciferases have also been employed and the functional expression of the whole Lux operon in Mtb and *M. smegmatis* allowed the development of auto-luminescent mycobacteria (Andreu *et al*. 2010). Luciferase-based bacterial replication are useful for monitoring Mtb replication in bulk but do not allow for single cell studies in cell-based models of infection (Fig. 7).

On the other hand, live cell studies allow for spatiotemporal resolution at the single cell level (Fig. 7). In some of these studies, that are rather limited, specific cellular environments that are favourable for Mtb replication were defined (Lerner *et al*. 2017; Mahamed *et al*. 2017). In these studies, it was observed in real time that once Mtb induced host cell death, it was able to rapidly grow inside the dead infected macrophage regardless of the differentiation or activation status. Replication inside dead cells was significantly faster than in the extracellular environment and once host cell death occurred, other macrophages internalized the dead infected cells, and this promptly led to their own death, amplifying the cell death response. Once there is substantial bacterial replication, correlative approaches were of great advantage to precisely define subcellular compartments permissive for bacterial replication such as necrotic cells (Lerner *et al*. 2017).

Alternatively, fluorescent reporters and probes can be used in order to monitor Mtb replication in host cells. One strategy used to monitor replication and phenotypic variation at the single-cell rates of rRNA transcription in Mtb is using a destabilised variant of GFP and a plasmid expressing the stable red fluorescent protein DsRed2 (Manina, Dhar and McKinney 2015). Using this reporter strain, and quantitative time-lapse microscopy combined with a genetically encoded fluorescent reporter (rRNA-GFP_des_) it is possible to investigate the single-cell dynamics of Mtb replication and rRNA expression *in vitro and in vivo*. These studies revealed a wide range of phenotypic heterogeneity even in bacteria grown under nutrient-rich conditions *in vitro* and increased heterogeneity in bacteria exposed to diverse stresses. For instance, stationary-phase populations showed a drop in rRNA-GFP_des_ expression followed by the appearance of a subset of non-replicating bacteria displaying high levels of fluorescence (Fig. 7). These observations confirmed that stationary-phase bacteria maintain a high level of *de novo* protein production, in agreement with studies in other bacteria (Rosenberg *et al*. 2012; Gefen, Fridman and Ronin 2014; Fridman *et al*. 2014). This reporter has been used mostly *in vitro* but one study also investigated mycobacteria in infected macrophages in drug screening studies looking for compounds that affect intracellular bacteria growth (Sorrentino *et al*. 2016). One of the caveats of this system is the low fluorescent signal from GFP_des_ and strategies using GFP_des_ in tandem could potentially increase signal to noise levels.

Another replication reporter has been developed and used to study the effect of vaccination on the Mtb replication status, at the single bacillus level. This reporter is based on an Mtb Erdman strain (SSB-GFP, *smyc*′::mCherry), consisting of a fusion of single stranded binding protein (SSB) to GFP, present on a replicating plasmid containing a constitutively expressed mCherry (Sukumar *et al*. 2014) (Fig. 7). Time point-based experiments with the Mtb (SSB-GFP, *smyc*′::mCherry) reporter strain allow visualisation of the heterogeneity in replication status within the bacterial population at the level of the individual bacterium in mice.

#### Spatiotemporal studies

In most of the cases time courses are performed with multiple fixed-samples at pre-defined timepoints. However, it is clear that some of the interactions are transient and dynamic. In order to unambiguously define the subcellular sites of Mtb replication/restriction in human macrophages and define whether Mtb can replicate in multiple cellular compartments, including lysosomes, continuous live cell imaging is crucial. Nonetheless, there are important obstacles in the analysis of the spatiotemporal organelle dynamic interactions with Mtb and the development of reliable methods to measure bacterial replication: (1) macrophage motility makes imaging problematic, (2) infection is rarely 100% synchronised making it difficult to capture statistically relevant numbers of events starting from time zero (time of bacterial entry), (3) macrophages die and aggregate at the site of bacterial replication and several processes of cell-to-cell transfer can dramatically interferes with the analysis and (4) the infection rates of macrophages are highly heterogeneous.

In recent years, several imaging approaches have been established to study the spatiotemporal detection of Mtb (Schnettger *et al*. 2017) and other mycobacteria (Delince *et al*. 2016) at the single cell level during *in vitro* infection. This represents an opportunity to study in more detail the precise roles of the different cellular pathways and their interplay with intracellular organelles for deciding the fate of Mtb in human macrophages.

### Concluding remarks: Mtb survival and replication in space and time

**Figure 8.**
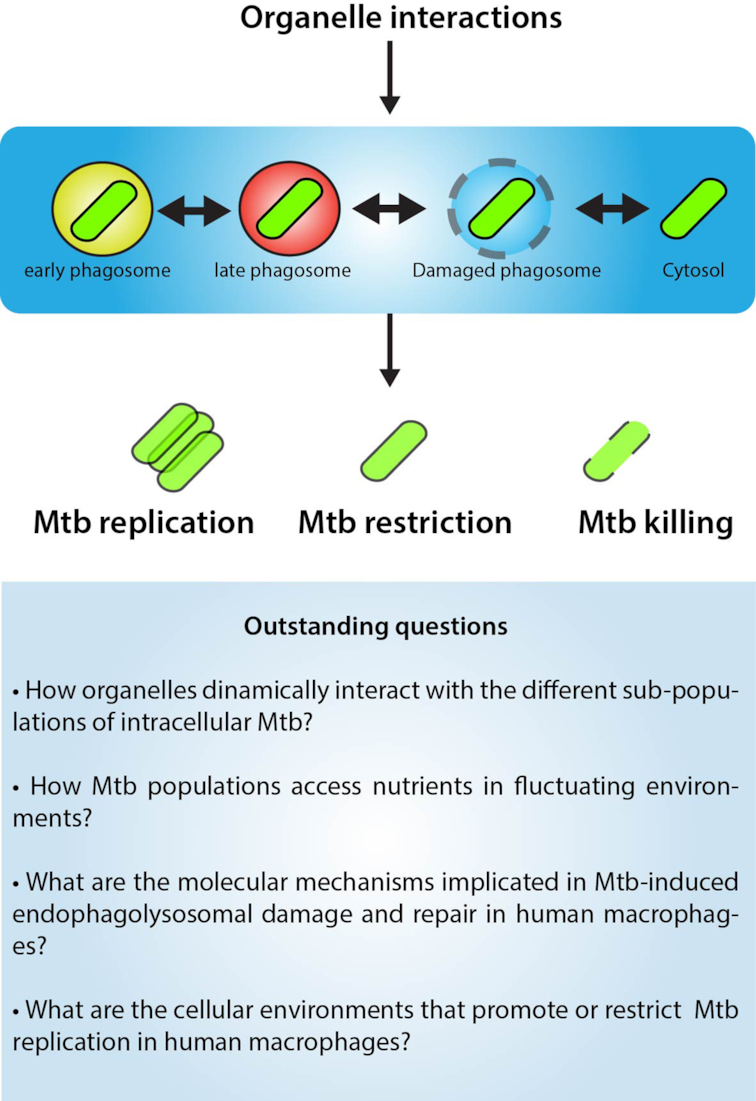
Spatiotemporal regulation of Mtb replication or control. In host cells, Mtb initially resides inside phagosomes where bacteria subvert phagosomal function. Some Mtb will effectively be targeted to late phagosomes where bacteria will be exposed to an acidic and proteolytic environment. At some point during the infection cycle, Mtb will damage phagosomes and access the cytosol. During these events, bacteria-containing compartments and cytosolic bacteria is actively interacting with a plethora of host cell organelles. The interactions between the different Mtb populations and host cell organelles will determine if Mtb replicates, or its growth is restricted or eventually eliminated by the host cell. Outstanding questions are shown emphasizing central themes in the cell biology of Mtb-host cell interactions requiring further research.

The precise cellular mechanisms that control Mtb replication in human macrophages, and in particular the mechanisms by which Mtb usurps the anti-bacterial host cell defence system, have not been completely elucidated. It is likely that the differentially localised populations of intracellular Mtb dynamically interact with cellular organelles during infection (Fig. 8). Spatiotemporal interactions between distinct Mtb populations and cellular organelles will dictate whether Mtb replicates, its growth restricted or eventually killed (Fig. 8). Given this background, there is a need to functionally define the precise locations where Mtb remains in host cells and their contribution to TB pathogenesis. In parallel, it will be important to identify the bacterial factors that allow Mtb to survive and eventually replicate in host cells and tissues.

The scarcity of knowledge in certain areas of host cell-Mtb interactions is likely a consequence of a lack of technologies that allow spatiotemporal resolution at the single cell level. For example, although the long-standing dogma in the field states that the lysosomal environment is detrimental for Mtb replication, several studies with mycobacteria challenge the notion that lysosomes are the sites where bacterial killing occurs (Armstrong and Hart 1975; Jordao *et al*. 2008; Levitte *et al*. 2016). There is no clear correlation between Mtb delivery to phagolysosomes and control of bacterial replication. A previous study showed that during Mtb replication in human macrophages, the number of bacteria in membrane compartments positive for CD63 (which is a frequently used marker of fusion of phagosomes with late endosomes and lysosomes)- or LAMP-1-positive phagosomes increased, suggesting that Mtb was able to replicate within compartments that have acquired the markers expected for lysosomes (Welin *et al*. 2011). Additional reports also showed that a proportion of Mtb was positive for Rab7 and other late endocytic markers (Seto *et al*. 2009; Harriff *et al*. 2014). However, heterogeneity and lack of single cell studies made it difficult to define where Mtb replicates. For instance, it is not possible to exclude that Mtb replicated in a non-fusogenic phagosome and consequently had been taken up into an autophagosome that has matured. Interestingly, the transmembrane serine protease MarP, which was identified in a screen for acid tolerance determinants, is also required for virulence (Vandal *et al*. 2008; Small *et al*. 2013). This is consistent with the idea that Mtb experiences acid stress *in vivo* and that the pathogen possess factors to counteract an acidic environment (Vandal *et al*. 2008).

From a therapeutic perspective, the intracellular lifestyle of Mtb represents a crucial stage in the disease and successful drug discovery programmes have to include *in vitro* studies using infected human host cells (Young *et al*. 2008; Lechartier *et al*. 2014; VanderVen *et al*. 2016); this *in cellulo* approach is now accepted as being more relevant than classical screening, that is based only on bacteria *in vitro*.
